# Use of consumer wearables to monitor and predict pain in patients with sickle cell disease

**DOI:** 10.3389/fdgth.2023.1285207

**Published:** 2023-10-25

**Authors:** Caroline Vuong, Kumar Utkarsh, Rebecca Stojancic, Arvind Subramaniam, Olivia Fernandez, Tanvi Banerjee, Daniel M. Abrams, Karin Fijnvandraat, Nirmish Shah

**Affiliations:** ^1^ Department of Pediatric Hematology, Emma Children’s Hospital, Amsterdam UMC, University of Amsterdam, Amsterdam, Netherlands; ^2^ Department of Engineering Sciences and Applied Mathematics, Northwestern University, Evanston, IL, United States; ^3^ Division of Hematology—Duke Sickle Cell Comprehensive Care Unit, Department of Medicine, Duke University Hospital, Durham, NC, United States; ^4^ Department of Computer Science & Engineering, Wright State University, Dayton, OH, United States

**Keywords:** sickle cell disease, pain, prediction, machine learning, ehealth, wearable

## Abstract

**Background:**

In sickle cell disease (SCD), unpredictable episodes of acute severe pain, known as vaso-occlusive crises (VOC), disrupt school, work activities and family life and ultimately lead to multiple hospitalizations. The ability to predict VOCs would allow a timely and adequate intervention. The first step towards this ultimate goal is to use patient-friendly and accessible technology to collect relevant data that helps infer a patient's pain experience during VOC. This study aims to: (1) determine the feasibility of remotely monitoring with a consumer wearable during hospitalization for VOC and up to 30 days after discharge, and (2) evaluate the accuracy of pain prediction using machine learning models based on physiological parameters measured by a consumer wearable.

**Methods:**

Patients with SCD (≥18 years) who were admitted for a vaso-occlusive crisis were enrolled at a single academic center. Participants were instructed to report daily pain scores (0–10) in a mobile app (Nanbar) and to continuously wear an Apple Watch up to 30 days after discharge. Data included heart rate (in rest, average and variability) and step count. Demographics, SCD genotype, and details of hospitalization including pain scores reported to nurses, were extracted from electronic medical records. Physiological data from the wearable were associated with pain scores to fit 3 different machine learning classification models. The performance of the machine learning models was evaluated using: accuracy, F1, root-mean-square error and area under the receiver-operating curve.

**Results:**

Between April and June 2022, 19 patients (74% HbSS genotype) were included in this study and followed for a median time of 28 days [IQR 22–34], yielding a dataset of 2,395 pain data points. Ten participants were enrolled while hospitalized for VOC. The metrics of the best performing model, the random forest model, were micro-averaged accuracy of 92%, micro-averaged F1-score of 0.63, root-mean-square error of 1.1, and area under the receiving operating characteristic curve of 0.9.

**Conclusion:**

Our random forest model accurately predicts high pain scores during admission for VOC and after discharge. The Apple Watch was a feasible method to collect physiologic data and provided accuracy in prediction of pain scores.

## 1. Introduction

Sickle cell disease (SCD) is the most common severe red blood cell disorder affecting 20 million individuals worldwide (1). In SCD, a mutation in the β globin gene leads to the formation of sickle hemoglobin (HbS). Deoxygenated HbS polymerizes into long chains changing the shape of red blood cells into a stiff, rigid and sickle shaped form. These sickled red blood cells can obstruct the microvasculature easily resulting in acute and chronic complications such as vaso-occlusion, hemolytic anemia, and multi-organ damage (2). Due to its broad range of complications, SCD is associated with increased morbidity, premature mortality, and impaired health-related quality of life (3, 4).

The most common complication of SCD are recurrent, acute episodes of severe pain, also called vaso-occlusive crises (VOCs). VOCs are the manifestation of vaso-occlusion along with tissue infarction, ischemic-reperfusion injury, and inflammation (5, 6). VOC pain is often located in the back, abdomen, or extremities, but any part of the body may be affected. A VOC usually lasts for 7 days and is often preceded by a prodromal phase of 1–2 days (7). VOCs may be elicited by dehydration, fever, cold temperatures, exertion, lack of sleep and stress. They usually occur unexpectedly and form an unwanted interruption of planned activities of the person with SCD. The unpredictability of VOCs profoundly affects school or work activities and family life.

Currently, treatment of VOCs begins at home and is focused on symptomatic pain control with hydration and analgesia. In case home management fails, evaluation within the emergency department and subsequent hospitalization for administration of opioids is often required. VOCs account for over 70% of acute care visits (8), and are the primary cause of hospitalization in approximately 95% of admissions of patients with SCD (9). Recurrent VOCs may progress to SCD-related chronic pain (10), as the prevalence of chronic pain increases with age. Chronic pain refers to pain that is present on most days for at least 6 months (11). By adulthood, over 55% of patients experience pain on greater than 50% of days (12).

Currently, there are no reliable tests to diagnose or predict VOCs in individuals with SCD. The gold standard for pain assessment and diagnosis is self-reported, leading to healthcare providers interpretation of pain reports, patient presentation, and medical history. Further, pain assessment tools such as the visual analog scale are limited by the momentary assessment of pain. There are laboratory parameters that have been associated with the severity of a VOC (13, 14), but they do not predict a VOC before it occurs (15, 16). Prediction or early recognition of pain is crucial, as it would potentially allow a timely intervention that possibly shortens a VOC and the development of complications. This unmet need to predict a VOC before it occurs may be approached by using mobile health applications that provide the opportunity to continuously monitor changes in physiological parameters. In order to develop clinical applications of VOC prediction by physiological parameters collected through mobile health applications, we need to first establish the feasibility of mobile monitoring in the outpatient setting and also refine the development of machine learning models to predict momentary pain scores.

Our recent efforts described by Stojancic et al, detailed our development of a machine learning model that was able to predict pain scores in SCD patients hospitalized for a VOC with an accuracy of 86% (17). However, during hospitalization the pain scores are expectedly higher than after discharge, when the pain subsides, scores are lower as they return to baseline levels. Therefore, to address prediction of pain scores in both the inpatient and outpatient setting, the present study aims to: (1) to evaluate the feasibility of extended monitoring up to 30 days after discharge from the hospital, and (2) to refine the development of machine learning models to predict pain scores.

## 2. Methods

### 2.1. Data collection

In this prospective cohort study, patients with SCD aged 18 years and above, who received care at Duke University Hospital, were eligible for enrollment. Patients were included if they were admitted for a VOC to the SCD day hospital or to Duke University Hospital between April and June 2022. The study protocol was approved by the institutional review board of Duke University Medical Center (IRB Pro00068979) and was conducted according to the Declaration of Helsinki. Following written consent, participants were enrolled for the duration of their hospitalization, and up to 30 days after discharge. They were provided: (1) the mobile app (Nanbar Health) on their personal Apple iPhone or provided with an iPhone series SE; and/or (2) an Apple Watch series 3 if patients did not have their own. Participants were instructed to report their pain score at least once daily in the Nanbar Health app. They were also asked to wear the Apple Watch as often as possible, removing it only to charge. The study team contacted the participants once a week by telephone or email to remind them to wear the Apple Watch and to report in the Nanbar Health app.

### 2.2. Study measures

Demographics including age, sex, SCD genotype, and ethnicity were collected from the electronic medical records (EMR). Details from the hospitalization were also collected from the EMR including pain scores reported to nurses. During hospitalization for VOC, pain scores were reported to nurses several times a day, and documented in the EMR. Self-reported pain scores were reported in the Nanbar app on a visual analog scale ranging from 0 to 10, with 0 accounting for no pain, and 10 being the most intense pain. Physiological data collected from the Apple Watch included heart rate, heart rate variability, average heart rate, resting heart rate and step count. Heart rate was collected by the Apple Watch every 3–7 min in rest, and periodically more frequently based on the activity level of the participant (18). Heart rate variability was calculated by using the standard deviation of beat-to-beat measurements that were captured by the heart rate sensor of the Apple Watch. Daily resting heart rate was calculated while inactive by the Apple Watch by correlating background heart rate readings with accelerometer data (18). These data were analyzed for association with the pain scores collected via the app and from EMR. The performance of the machine learning models was evaluated using the following metrics: accuracy, F1-score, area under the receiving operating characteristic curve (AUC) and root-mean-square error (RMSE) (Table 1) (23). F1-score was calculated using precision and recall. Refer to the Supplementary Table S1 for the formulas. Calculation of AUC was done for each class and the average value was reported.

**Table 1:** Definition table of the used metrics to evaluate the performance of each model.

Accuracy (19)	The proportion of correct data points predicted by the machine learning algorithm out of all the data points.
F1-score (20)	Considers not only the accurate recall of a model but how close together predicted values are to each other (“1” is considered a perfect model).
Area under the receiving operating characteristic curve (AUC) (21)	Determines how well our model picks between different pain score classes. In our model, each numerical pain score is a class (“1” is considered a perfect model).
Root-mean-square error (RMSE) (22)	Refers to how far the true values are from values predicted by our model. Larger values represent the further distance between predicted and true values.

We also compared the machine learning models to two null models, random model, and mode model. These models do not take in any physiological data to train, rather use the frequency information of each pain score in the training data set to define a rule. Random model assigns probability of each pain score to occur proportionate to their frequency in the training data. On the other hand, the mode model predicts the pain scores to be the equal to the most frequent pain score in the training data. These null models have no clinical significance but are useful to assess the validity of the created machine learning models as their predictive value can be compared to the results of these models based on chance. Null models also help establish a baseline against which we assess performance of ML models. The physiological data was collected through the Apple Watch. However, due to infrequent self-reporting of pain on the app, we combined these self-reported pain scores with the pain scores from the EMR. Physiological data were solely collected by the Apple Watch.

### 2.3. Analyses

All data were analyzed using Statistical Package for the Social Sciences (version 28.0; SPSS) or Python (version 3.9.6; Python Software Foundation). Descriptive data was generated for all variables to describe the study population. Categorical variables were presented as absolute numbers with corresponding percentages. Means and standard deviations (SDs) were calculated for continuous variables that were normally distributed. Medians with interquartile ranges [IQRs] were calculated for values that were not normally distributed. The submitted pain scores were associated with the physiological data using the nearest-neighbor approach. As the pain scores were discrete pain values ranging from 0 to 10, three classification machine learning models were fit to our data: multinomial logistic regression, random forest, and gradient boosting model. The hyperparametric values for all the models were set to be the default value set in the scikit-learn library for Python. Post the initial fit, we tuned the maximum depth of individual trees in the random forest model, to avoid overfitting and underfitting. To increase the amount of data for the machine learning models, we adopted an oversampling scheme, where it was assumed that the pain score remained the same for up to 15 min prior to and after each pain score was recorded. The metrics chosen to showcase the performance of each model were accuracy, F1-score, area under the ROC curve (AUC) and root mean squared error (RMSE). Out of these metrics, accuracy and F1-scores were micro-averaged. Micro-averaging is a method used to calculate certain performance metrics which enables us to consider each data point individually and not individual classes. Using this method helps avoid minority classes from skewing the metrics and gives us a more realistic reflection of the model performances. For the best performing machine learning model, we calculated a feature importance score for the physiological variables to determine which variable contributed the most to the prediction of the machine learning model. To further assess the model performance, instead of the random split approach, we used 10-fold cross-validation and presented the mean accuracy and its standard deviation (SD). Cross-validation allows us to make use of all the data by dividing it in equal parts (or “folds”) and then train and test the models on different folds on different iterations. The accuracies are reported using the average value with the standard deviation over all 10-folds. It is important to note that the average is the unweighted mean (macro-average), and not micro-averaged.

## 3. Results

Our study included 19 patients with SCD with the median age of 30 years [interquartile range (IQR 22–34)], of which the majority had HbSS. The demographics are presented in Table 2. The median length of stay of the hospitalized participants was 5 days [IQR 2–9.8]. All patients were treated with opioids. During hospital admission, no patients developed an acute chest syndrome, and only 1 patient required oxygen support. Within the 30 days post-discharge period, 14 participants received subsequent medical care for pain (74%). There was no collinearity between any of the data elements recorded by the Apple Watch.

**Table 2:** Demographics of the included participants.

Participants	Median [IQR] or *n* (%)
Age (years)	30 [IQR 22–34]
% Female	11 (58%)
African-American	19 (100%)
SCD genotype	
HbSS	14 (74%)
HbSC	5 (26%)
Place of enrollment	
Hospital	10 (53%)
Day Hospital	9 (47%)
Time wearing watch (days)	28 [IQR 22–34]

IQR, interquartile range; n, number.

### 3.1. Dataset

**Figure 1:**
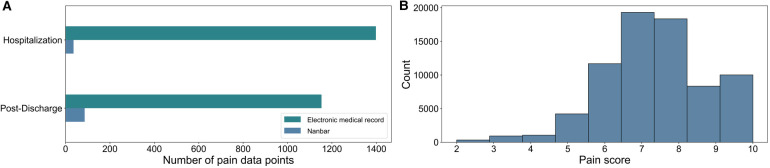
(**A**) Split between the size of electronic medical record (EMR) data and Nanbar data before oversampling. We had 2,395 data points after combining the pain scores from the application, and those recorded in the EMR. (**B**) The final distribution of reported pain scores numerical values after oversampling, where pain scores were assumed to remain the same for 15 min prior to and after each pain score was recorded.

The median number of pain data points per participant was 79 [IQR 16–142]. After combining the pain scores from the app and those recorded in EMR, our dataset consisted of 2,395 pain data points. In our dataset, there were no 0s or 1s reported, therefore we used 9 classes in which each class represented pain scores ranging from 2 to 10. In Figure 1A, the number of pain data points are shown for hospitalization and the period after discharge; 2,273 pain data points were derived from the EMR, and 122 data points came from the Nanbar app. The distribution of the reported pain scores after oversampling are shown on a scale from 0 to 10 in Figure 1B. The pain score seven was the most frequently reported in our dataset.

### 3.2. Performance of models

**Figure 2:**
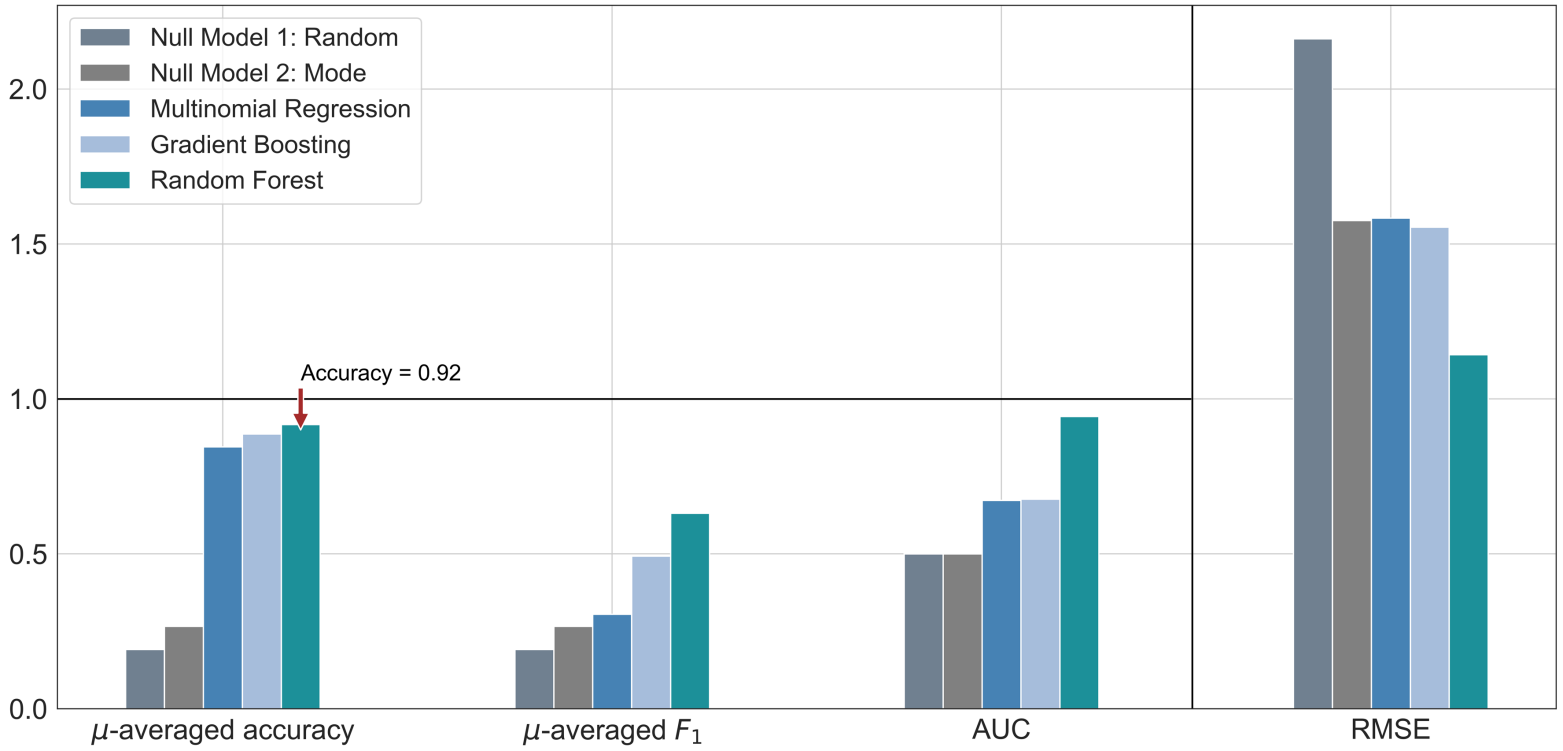
Bar graph comparison of the evaluation metrics for each machine learning model, along with two null models. These were calculated from a single random split of the dataset. The symbol µ is being used to represent the word “micro”. Accuracy and F1-score were micro-averaged. The horizontal black rule indicates the upper bound for accuracy, F1 score and AUC, the extended scale only applies to RMSE. AUC, area under the receiver operating characteristic curve; RMSE, root-mean-square error.

**Figure 3:**
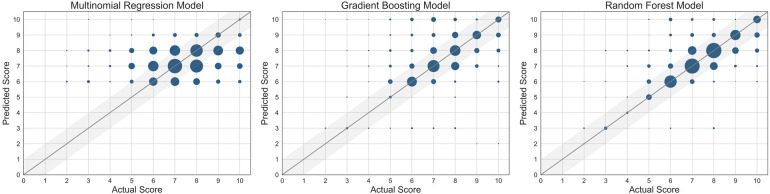
Scatter plots of the three machine learning models used. The size of the marker is proportional to the number of data points at the same grid point. The straight line represents where the predicted score = actual score.

**Figure 4:**
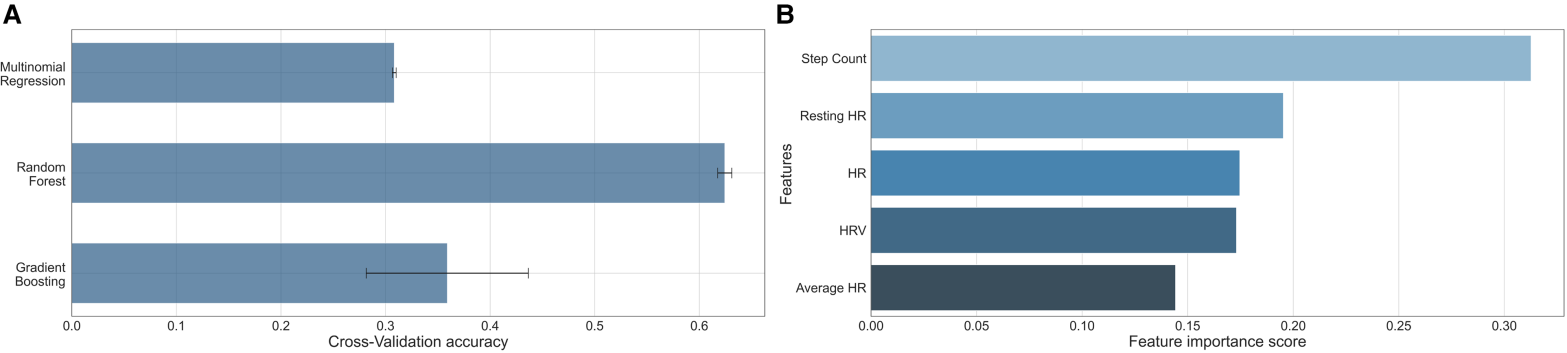
(**A**) Bar graph comparison of the 10-fold cross-validation accuracy of the three machine learning models. The reported value is the macro-average (numerical mean) and the error line is the standard deviation of the accuracies achieved across all 10-folds. (**B**) Bar graph comparison of the importance of the features showing step count was the most important. HR, heart rate; HRV, heart rate variability.

The performance of each machine learning model is shown in Table 3. F1-scores were calculated using precisions and recalls, which are reported in the Supplementary Table S2. As demonstrated in Figure 2, all three machine learning models outperformed the null models. The random forest model was the best performing machine learning model based on the metrics, as shown in Table 3 and Figure 3 with an accuracy of 92%, micro-averaged F1-score of 0.63, AUC of 0.9, and a RMSE of 1.1. The cross-validation accuracy and SD of the three machine learning models are shown in Figure 4A. The cross-validation demonstrates that the random forest model had the highest cross-validation accuracy (62%) with the lowest SD (0.7%). These cross-validation accuracies are macro-averaged averaged and hence get skewed by the class imbalance. However, regardless of the difference in macro and micro-averaged accuracies, these values indicate that this model is most likely to outperform the rest of the models in an independent dataset in terms of both performance and robustness.^1^ Among the five physiological variables used in the random forest model, step count had the highest feature importance score followed by resting heart rate as shown in Figure 4B.

**Table 3:** The performance of each model including the two null models.

Prediction model	µ-averaged accuracy (%)	µ-averaged F1-score	AUC	RMSE
Null model 1: random	19.7	0.20	0.5	2.2
Null model 2: mode	26.7	0.27	0.5	1.6
Multinomial regression model	84.6	0.31	0.7	1.6
Gradient boosting model	87.0	0.41	0.7	1.5
Random forest model	91.9	0.63	0.9	1.1

µ, micro; AUC, area under the receiving operating characteristic curve; RMSE, root-mean-square error.

## 4. Discussion

Physiological data can be feasibly collected by a consumer wearable and be used to build machine learning models that successfully predict self-reported pain scores in patients with SCD admitted for VOC and up to 30 days after discharge. Despite the fact that the random forest model is not the most interpretable machine learning model, it did outperform the other machine learning models. The random forest model was most accurate in the prediction of the higher pain scores, particularly those greater than 6. The random forest model had also the highest cross-validation accuracy. Accurate pain prediction is key in the management of a VOC, as it is often depending on how much pain the patient is experiencing.

### 4.1. Related works

These findings are in line with the previous studies performed with patients during treatment for VOC, while admitted to the day hospital (17, 24). In the study from Stojancic et al, the random forest model was also the best performing machine learning model (17). Although our machine learning model achieved a slightly higher accuracy (92% vs. 85%), the model from Stojancic et al. performed considerably better given the other metrics such as the F1-score (0.63 vs. 0.85). Even though data collection continued after discharge in our study, we found a high reutilization of care rate 30 days post-discharge indicating that a substantial proportion of the patients were still in pain during follow-up after the hospitalization. It is also possible that patients who were feeling well after discharge did not feel the need to continue to report if they had no or low levels of pain, introducing an additional bias towards higher pain score reporting. The class imbalance was less pronounced in the study by Stojancic et al, because of the regular pain reporting to nurses reflected by the higher F1-score. Also, the Apple Watch was put into exercise mode during the previous study, which allowed continuous data collection with data acquisition every minute. This was not feasible in our outpatient study due to the resulting shortened battery life. All the above contributed to a higher data density and a better performing machine learning model in Stojancic et al.

Another study that focused on the prediction of acute pain in patients with SCD presenting to the day hospital was conducted by Johnson in 2019, but with the Microsoft Band 2. Similar machine learning analyses were performed showing an accuracy of 73% on a 4-level pain score with the machine learning model the support vector machines for regression (24). In this study, 27 patients were included at the day hospital for the average duration of 3.8 hours. In contrast to previous studies at the day hospital, we had a higher accuracy most probably because of the longer duration of follow-up outside the hospital, the applied machine learning techniques, and the improved technology of wearable devices over time.

Instead of using physiological data from a wearable device, Panaggio et al. used physiological data from medical records for estimating pain in 46 patients with SCD (25). They used two probabilistic classification models, and used three classes to approach the pain level (low, medium, high). Similar to our study, their machine learning models outperformed null models, showing that physiological measures should be used to infer subjective pain levels and changes in pain levels. However, our best model performed better on multiple metrics compared to the best model in Panaggio et al.

While within the walls of the hospital, the prediction of pain works sufficiently, there have been other efforts that used technology to understand SCD-related pain outside the hospital. In the study conducted by Fischer et al, data was collected from an actigraph device during sleep in a study among children with SCD (26). They found that worse sleep efficiency was associated with the next-day pain and more severe pain (26). In another study by Ji et al, the authors used finger photoplethysmogram and heart rate measured overnight to successfully build a machine learning model that was able to predict future VOCs by correlating peripheral vasoconstriction to experiencing future VOCs in patients with SCD (27).

Interestingly, in our study, step count was found to be the most important feature in the random forest model. In the ELIPSIS study, the authors also found a statistically significant reduction in average daytime activity during VOC compared to the days without pain in the home setting (28). In Tsai et al, daily step count was a unique predictor for pain intensity and pain interference in patients with chronic pain (29). This potentially can be explained by the fact that patients who were in pain are often less active due to the pain (30). The second most important feature we found for prediction of pain was resting heart rate. Physiologically, acute pain is associated with a stress response, increasing the heart rate (31). Although four out of five features were derivatives of heart rate, all features were not correlated with each other. In our machine learning model, heart rate variability was the fourth most important feature. Previous research shows that heart rate variability is a more sensitive marker of stress, compared to resting heart rate (32). Changes in heart rate variability, a marker for autonomous nervous system, have been found to be associated with a VOC. For example, in the study by Adebiyi et al, heart rate variability was found to be significantly different between patients with SCD during a VOC and patients in steady state (33). Stress reduces the heart rate variability and previous research in patients with diabetes mellitus type 1 has demonstrated that reductions in heart rate variability precede hypoglycemia by hours (34). Similarly, VOCs in patients with SCD may also be identified by changes in vital parameters preceding a VOC.

It should be recognized that we only evaluated five features in our machine learning model, therefore other possibly relevant physiological features that can be measured by a wearable device, such as sleep, should be considered as well as in future feature selection. Although prediction of pain score using wearable device is the first step, the ultimately goal is to detect deviations of patterns in physiological data indicative for VOC as described by the anomaly detection framework (35). Correlating physiological data to the prodromal phase of a VOC may allow for early patient notification of an upcoming VOC, and timely administration of medication and fluids. This could potentially reduce the severity of the VOC, avoiding the need of a hospitalization, and the development of complications which occur during VOCs, such as the acute chest syndrome. We believe that wearable devices, including consumer wearables, have the ability to increase the availability of personalized healthcare to underserved communities. In the last decade, wearable devices have advanced rapidly, are more readily available and accessible, which increases the opportunities for remote caregiving. Access to this type of information may allow patients to be remotely monitored and managed, a concept being adapted for other chronic diseases. Continual availability to personalized health information can increase awareness and decrease the amount of time patients spend admitted in a hospital thereby increasing their health-related quality of life and decreasing the cost of care. The ability of machine learning algorithms to predict the occurrence of pain with real-time wearable data allows a more personalized treatment in the management of pain in SCD. Other advancements with machine learning in SCD are summarized by Elsabagh et al. (36).

### 4.2. Strengths and limitations

Study strengths include the follow-up period of the study after discharge from the hospital, as patients are often not entirely free of pain when discharged. Data collection continued in the home setting for 30 days, while recovering from the VOC, and leading to more pain scores from participants not in significant pain. This allowed us to collect a higher variety of pain scores compared to our previous studies, that were conducted within the hospital. By combining the data from the Apple Watch, Nanbar application and the EMR, we were also able to create an optimal and personalized machine learning model for the prediction of the higher pain scores, comparable to the real-life setting and applicable to outside the walls of the hospital. We expect that as the use of wearable devices becomes more common, such as the use of fitness trackers during exercise, patients will wear and report more often for their disease as well, improving the machine learning models.

Even though we combined the collected data from multiple sources, the leading limitation of the study stems from the relatively small number of self-reported data points within the mobile app. For this reason, we could not perform sub-analyses stratifying for SCD genotype or type of pain (acute, daily and chronic). However, our study is a first step towards digitalizing the complete process of data collection and management. Moving in this direction would lead to taking away the reliance on data acquired only in-person and potentially pave the way for machine learning applications for pain prediction. Future efforts should focus on providing more information about the physiological data in steady state, as machine learning models perform better with higher variety of and larger quantities of data. Nonetheless, the performance of the machine learning models for pain prediction in our pilot study was satisfactory and promising given the small number of patients. Furthermore, we believe future efforts with data on medication could significantly improve the pain prediction as shown in the study by Padhee et al. (37).

In future studies, to ensure that the participants remember to report pain and symptoms on a regular basis, which is important for a robust data collection, we will use daily push notifications within the app to remind participants to report and strategies such as a badge reward system. Additionally, routinely quality checks on the patient-reported data through a dashboard to confirm that patients are logging regularly without technical issues should improve the quality of the data. By allowing participants to use their personal devices, the quantity of data collection should improve and has led to efforts to build an Android version of the Nanbar Health app.

## 5. Conclusion

Consumer wearable devices such as the Apple Watch are useful, non-invasive, and patient-friendly methods for continuous data collection in- and outside the hospital. They are a valuable source of data for machine learning analyses and show promise in accurately predicting pain. This type of healthcare can benefit both patients with SCD and clinicians, enabling early detection of VOC and timely intervention by providing personalized health care. Given the increased accessibility of technology worldwide, the use of mobile health may be able to transform healthcare in rural areas not only in a more convenient, but also in a more affordable manner.

## Data Availability

The raw data supporting the conclusions of this article will be made available by the authors, without undue reservation.

## Footnotes

^1^Our choice of method is not necessarily the best-performing one across all possible machine learning models but rather shows that we can use machine learning to predict pain with a fairly high degree of ‘accuracy’.
