# Supplementary material for: Use of consumer wearables to monitor and predict pain in patients with sickle cell disease
Source: Front Digit Health. 2023 Oct 25;5:1285207. doi: 10.3389/fdgth.2023.1285207 (PMC10634543; doi:10.3389/fdgth.2023.1285207)
Supplement: Supplementary file 1 [file Table1.docx]

***Supplementary Material***

**Use of consumer wearables to monitor and predict pain of patients with sickle cell disease**

**Caroline Vuong^1^*, Kumar Utkarsh^2^, Rebecca Stojancic^3^, Arvind Subramaniam^4^, Olivia Fernandez^3^, Tanvi Banerjee^5^, Daniel M. Abrams^2^, Karin Fijnvandraat^1^, Nirmish Shah^3^**

*** Correspondence:** Caroline Vuong: c.vuong@amsterdamumc.nl

# Supplementary Table 1

| **Metric** | **Mathematical formula** |
| --- | --- |
| Accuracy | $A= \frac{tp+tn}{tp+tn+fp+fn}$  Tp = true positive  Tn = true negative  Fp = false positive  Fn = false negative |
| Precision:  Recall:  F1-score | $P= \frac{tp}{tp+fp}$  $R= \frac{tp}{tp +fn}$  $F1=2* \frac{P*R}{P+R}$  Tp = true positive  Fp = false positive  Fn = false negative |
| Area under the receiving operating characteristic curve | $\frac{1}{2}\left( \frac{tp}{tp+fn}+\frac{tn}{tn+fp} \right)$  Tp = true positive  Tn = true negative  Fp = false positive  Fn = false negative |
| Root-mean-square error | $\sqrt{{\frac{1}{n}\sum_{i=1}^{n} (Y_{p,i}}-{Y_{a, i}}}){}^{2}$  Y_p,i_ is the predicted output  Y_a,i_ is the actual output. |

**Supplementary Table 1.** The formulas to calculate the metrics for the evaluation of the machine learning models.

# Supplementary Table 2

| **Model** | **Precision** | **Recall** |
| --- | --- | --- |
| Null model 1: Random | 0.20 | 0.20 |
| Null model 2: Mode | 0.27 | 0.27 |
| Multinomial Regression | 0.31 | 0.31 |
| Gradient Boosting | 0.41 | 0.41 |
| Random Forest | 0.63 | 0.63 |

**Supplementary Table 2.** Precisions and recalls of the machine learning models.

# Supplementary Data 3.

The code can be requested from Northwestern University’s online repository from the following link:

<https://doi.org/10.21985/n2-js7v-q051>.
